# Pathologic Features of Behçet's Disease in the Tubuler Gut

**DOI:** 10.1155/2012/216254

**Published:** 2011-11-15

**Authors:** Tuba Kara, Duygu Düşmez Apa

**Affiliations:** Department of Pathology, School of Medicine, Mersin University, 33010 Mersin, Turkey

## Abstract

Behçet's disease (BD) is a vasculitic disorder of relapsing acute inflammation characterized by recurrent oral ulcers, genital ulcers, uveitis, and skin lesions. The disease also affects other organs, including joints, the nervous system, blood vessels, and gastrointestinal (GI) system may also be involved and the lower GI tract is the mostly involved part, leading to severe morbidity. The frequency of GI involvement in BD varies among different ethnic groups. Although 50–60% of Japanese patients have GI disease, these manifestations are rare in patients from Mediterranean countries. The gastrointestinal manifestations of BD usually appear 4.5–6 years after the onset of the oral ulcers. The intestinal lesions are usually resistant to medical treatment and recur after surgery. The elementary lesion is apthous ulcer. Deep, round or oval ulcers with a punched-out appearance tend to perforate easily, so that many patients require urgent operation.

## 1. Introduction

Behçet's Disease (BD) is a chronic rheumatic disease, in which unpredictable inflammatory episodes of orogenital and ocular inflammation (or ulceration) are induced by the body's overreactive immune system, was first reported in 1937 by Hulusi Behçet. 

Patients with incomplete form of Behçet's syndrome without ocular involvement, according to Morita et al., had been well described in the Japanese population [1]. Countries with a high prevalence of BD cluster along the ancient silk road from Eastern Asia to the Mediterranean Basin. 

Young adults between the second and fourth decades of life are mainly affected [2]. In children, this type of BD occurs very rarely, usually as the perianal aphthous lesions that appear to be a specific feature of childhood BD [3]. 

Behçet's disease is more common and more severe in men than in women in the ancient Silk Road countries, whereas in Western Europe and the USA, it is equivalent between the sexes or more common in women [2]. Populations has yielded interesting epidemiological findings: Turkish individuals who have emigrated to Germany have a significantly lower risk of disease than individuals of Turkish origin living in Turkey, although their risk remains higher than that of the native German population. 

The most plausible environmental trigger is an infectious agent, for that reason evidence of viral infection has been sought. Increased rates of anti-Saccharomyces cerevisiae antibodies (ASCAs); which are recently proposed serologic markers of disease and commonly associated with Crohn's disease (CD), were previously reported by several studies in BD [4]. However these antibodies are present in 44% of BD patients who have intestinal involvement of the disease, compared with 3% in patients without gastrointestinal symptoms [5]. Alternatively, BD may be primarily autoimmune in origin [4]. Various genetic markers have been identified, especially regarding tumor necrosis factor-*α* (TNF-*α*) and various proinflammatory cytokine polymorphisms. Recent reports have focused on the TNF-∞ gene, which is closely linked to the HLA-B51 gene, in view of the major role played by this proinflammatory cytokine in BD. In the Turkish population, the TNF-∞1031C allele was associated with susceptibility to Behçet's disease [5]. 

Pathologically the disease is characterasized by vasculitis targeting the vasa vasorum and other small blood vessels, which is usually lymphocytic and affects veins more than arteries, although occasionally there may be more necrotizing inflammation with leucocytoclasis. Fibrinoid necrosis may be present in involved veins and venules [6]. There is intense inflammation around the vasa vasorum, resulting in destruction of the media and fibrous thickening of the intima and adventitia. Pseudoaneurysms are the most common arterial manifestations, mainly involving the aorta, the pulmonary, and femoral arteries [2]. While thrombophlebitis of the superficial and deep veins is common in BD, thrombosis and aneurysms of the large arteries in the abdominal cavity are rare but lead to death [7]. 

Diagnostic criteria of BD are defined by the1990 International Study Group for BD. Recurrent oral ulceration must be present along with two of the followings: genital ulcerations, typical eye lesions, typical skin lesions, or positive pathergy test [2–5].

## 2. Gastrointestinal Manifestations of Behçet's Disease

The gastrointestinal manifestations of BD usually appear 4.5–6 years after the onset of the oral ulcers [3]. The most frequently affected site in GI system is the ileocecal region, with extension into the ascending colon. Common clinical symptoms include abdominal pain, diarrhea, and bleeding, similar to those of inflammatory bowel disease [8]. The frequencies of GI involvement vary among different ethnic groups. In Japan and Korea, the prevalence of GI involvement is higher (50–60%), whereas in Turkey and Israel the prevalence is much lower (0–5%) [9, 10]. In the series of 2313 BD patients of Tursen et al. GI involvement was found in 31 patients (1.4%) [11]. Intestinal involvement in BD is rare but important because it is one of the most common causes of fatality, and severe morbidity during the course of the disease [9]. Intestinal BD can be diagnosed if a patient shows typical ulcer in the small or large intestine, and if systemic manifestations meet the diagnostic criteria of BD [12]. The genetic and environmental factors influence the frequencies, but the series so far reported included only small numbers of cases and were not standardized [13]. 

GI symptoms are often caused by nonspecific ulcers. While all patients develop oral lesions, it is the presenting manifestation in about 70% of patients [2]. However, these lesions are not pathognomonic of BD and can be seen in a variety of other clinical conditions such as anemia, avitaminosis, viral infections, inflammatory bowel disease, and Reiter syndrome. The oral ulcers of BD are aphthous or herpetiform, and occur at least three times a year in the absence of other clinical explanations for the ulcers [3]. The lesions are punched-out ulcers with rolled or overhanging edges and a necrotic base, surrounded by an erythematous rim. They are generally painful and heal with little scarring, with or without treatment [2]. Moreover, the recurrent oral ulcerations of BD tend to be multiple and usually involve the soft palate and oropharynx [3]. Smoking may decrease the severity of ulceration in BD. Histologically, there is vasculitis with an infiltration of monocytes and lymphocytes early in the disease and neutrophils in older lesions. Additional findings are fibrinoid necrosis, endothelial swelling, and a perivascular infiltrate in both early and late lesions [2].

Esophageal involvement is uncommon. However, upper GI involvement may be underestimated because endoscopic examination is not performed routinely in BD and there are few clinical trials investigating the upper GI tract in BD [14]. Since the first case of esophageal ulceration of this syndrome was presented by Brodie and Ochsner in 1973, to date, less than 50 cases of Behçet's patients with esophageal ulcers have been reported worldwide [15]. Houman et al. studied 23 patients with BD (four with upper GI symptoms) and found endoscopic, manometric, and/or microscopic abnormalities in 14 patients; histopathologic abnormalities were found in only five patients. They concluded that the prevalence of esophageal involvement in BD was high but these findings were not confirmed by other studies [14]. It usually involves the middle part of the esophagus, causing substernal pain, dysphagia, and occasionally hematemesis, although diffuse esophagitis and stenosis have also been reported [2, 15]. The esophageal ulcerations in BD can be single or multiple, and are often associated with ulcers in other parts of the gastrointestinal tract. Esophageal lesions reported to occur in patients with BD are (1) linear, oval, or round ulcerations similar to those that are present in the mouth, (2) ulcers or fistula that communicate with adjacent organs such as trachea, (3) luminal strictures, a pseudomembranous esophagitis, and (4) “downhill,” or classical, esophageal varices associated with either superior vena caval obstruction or portal hypertension due to portal vein thrombosis [3]. In addition, serious complications such as stricture, bleeding, or perforations have been rarely described [15]. Histology is nonspecific, showing lymphocytic or neutrophilic infiltration rather than vasculitis. The lesions may be resistant to treatment with a proton pump inhibitor but resolve with corticosteroids. Since BD is treated with immunosuppressive agents, viral, or candida esophagitis should be excluded [2].

The stomach is the least frequently involved part of the GI tract. An exception is the surprisingly high number of Taiwanese patients with BD (45%) who have gastric or duodenal ulcers [2]. Ning-Seng et al. found a close relationship between A2/B46/Cw1 (or A11/B46/Cw1) genotype and the development of gastric/duodenal ulcers [16]. Aphthous ulcers are the most common finding however there may be pyloric stenosis due to edematous hypertrophy of the pyloric ring or a Dieulafoy's ulcer [2]. Two studies assessing the frequency of *Helicobacter pylori* (*H. pylori*) infection in Turkish patients with BD have been reported with an urease positivity rate of 65% in the first and 85% in the second report and the presence of *H. pylori* correlated with the disease activity, manifested as the presence of gastrointestinal complaints or endoscopic findings [3]. In their study, Çakmak et al. suggest that the prevalence of upper GI abnormalities in BD is high and may occur in asymptomatic patients, but that abnormalities are not specific for BD. Therefore, routine endoscopy and screening for *H. pylori* infection may not be necessary in asymptomatic patients [17]. In one report, treatment of *H. pylori* diminished the oral and genital ulcers. Furthermore gastric pathogen *H. pylori* produces heat shock proteins which is thought to play a role in the etiopathogenesis of BD and may cause ulcers in the GI tract [18].

The intestinal lesions of BD occur in two forms: mucosal inflammation and ischemia/infarction. The clinical presentation of intestinal BD is similar to that of CD, which shares many extraintestinal features such as oral lesions, uveitis, and arthritis. The other stigmata of BD may appear later than the gastrointestinal features. Thus, in areas of the world where BD is common ileocolitis should be followed closely for the other major stigma of BD and should be assumed to be a manifestation of idiopathic inflammatory bowel disease [3]. 

**Figure 1:**
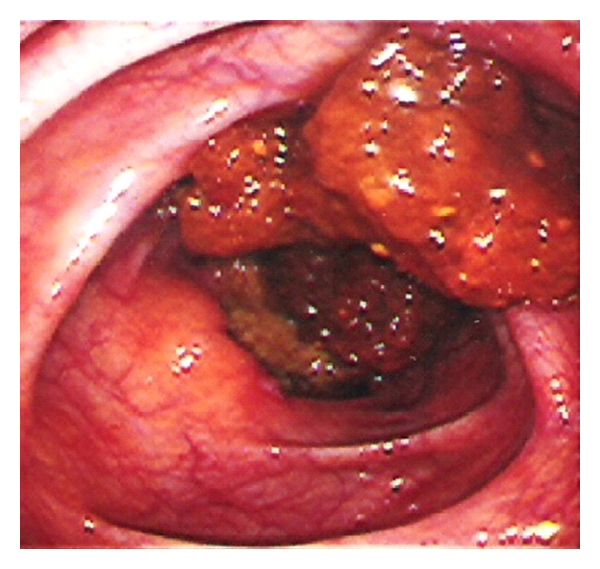
Endoscopically detected pseudopolips in small intestine mucosa (with permission of Dr. O Sezgin).

**Figure 2:**
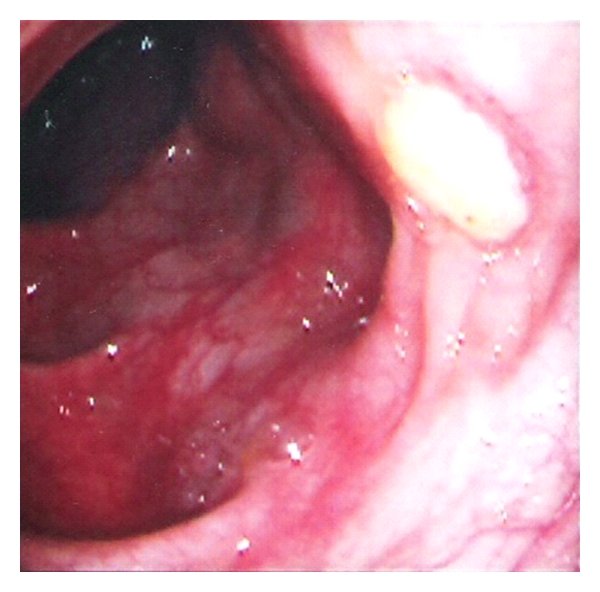
Endoscopically detected ulcers in small intestine mucosa (with permission of Dr. O Sezgin).

**Figure 3:**
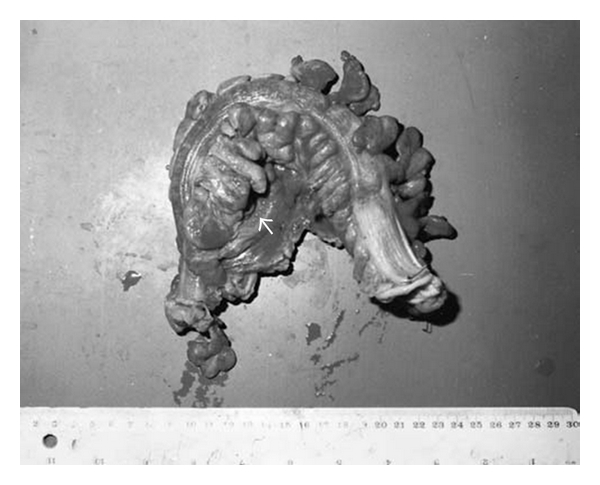
Macroscopic specimen showing hemorrhagic areas on the serosal surface and perforation (*arrow*) (with permission of Dr. DS Arici).

The small intestine is the most frequently involved extraoral part of the gastrointestinal tract in cases of BD. The lesions are most commonly found in the terminal ileum, particularly in the area of lymphoid aggregates and Peyer's patches, and the cecum, and less frequently in the colon. Rectal and anal involvement is quite rare. Intestinal lesions are located on the antimesenteric side [19]. Ileocecal involvement in cases of BD is common in Japan and Turkey, while colonic involvement is the usual pattern in Europe and North America [3]. Isolated involvement of the ileum is not uncommon in BD. Ileal visualization should be performed at colonoscopic examination in clinical practice to document the intestinal involvement. Most of the patients with BD have inflammation in the ileum, even in the absence of visible lesions [13]. The most common colonoscopic findings are localized single or multiple ulcers in the ileocecal region, with only 4% having a diffuse distribution of lesions (Figures 1, and 2). The ulcers may be aphthous or, alternatively, deep and round with a punched-out appearance. They are usually localized but multiple in number and may resolve with medical therapy. Longitudinal ulcers are rare. Fissuring and linear ulcers up to 5 cm long may also be present. Enlarging or newly formed ulcers coexist with healed ulcers. They have a tendency to irregularly undermine surrounding tissues. Edema-like swelling with crater formation around the ulcer margins produces a characteristic “collar-stud”appearance [20]. Adjacent to larger ulcers there are often smaller aphtoid ulcers. BD is particularly charecterized by adjcent macroscopically normal-appearing mucosa [6]. Multiple superficial ulcers located predominantly in the terminal ileum in Turkish patients differ from the single, large, deep ulcers with distinct borders described in the Far East. By the way, intestinal perforations may occur more commonly in Far East patients than West [12, 19, 21] (Figure 3) [22]. The exact perforation mechanism in intestinal BD is not clear, but there are several hypotheses. First, typical large, discrete, and excavated ulcers penetrate, resulting in perforation. Second, bowel dilatation may lead to perforation. Third, long-term steroid use may be associated with the development of bowel perforation. J. Chou et al. reported a series of 22 patients with multiple intestinal perforations [23]. 

**Figure 4:**
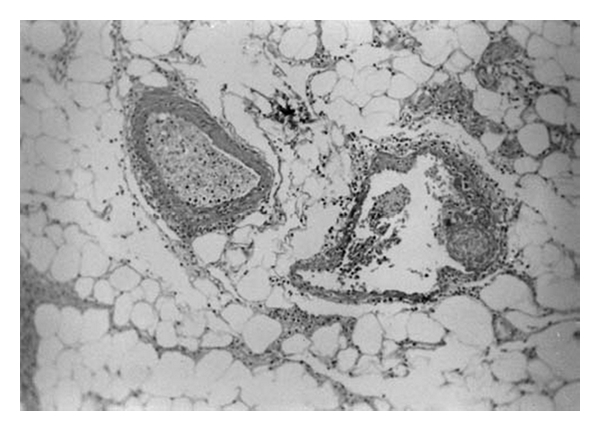
Histopathologic examination showed nonspecific inflammatory cell infiltration surrounding the vessels (HE ×25) (with permission of Dr. DS Arici).

**Figure 5:**
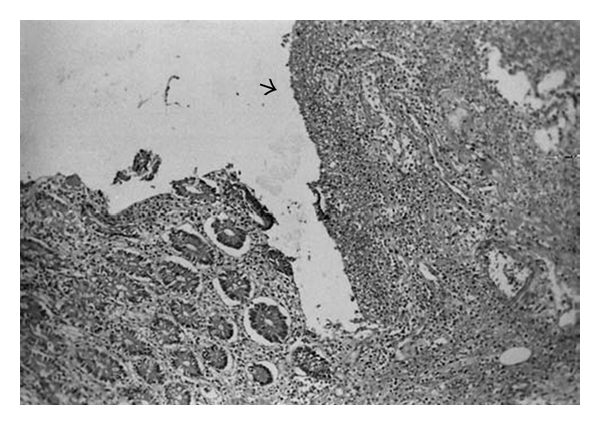
Mucosal ulceration (H&E ×10) (with permission of Dr. DS Arici).

The colonic ulcers in BD have been classified as volcano-type, geographic, and aphthous. Volcano-type ulcers were defined as well-demarcated deeply penetrating ulcers with nodular margins, converging folds, or pseudopolyps. Geographic-type ulcers were defined as shallow ulcers with sharp edges, while aphthous-type ulcers were small, punched-out shallow ulcers. Volcano-type ulcers had a less favorable response to medical treatment, more frequent requirement for surgery, and more frequent recurrences than the other two types [2]. Histologically, vasculitis of the small veins and venules is common in cases of intestinal BD. It is characterized by a lymphocytic infiltrate (Figures 4, and 5) [22]. However, chronic nonspecific (sometimes transmural and granulomatous) inflammation with a normal intervening mucosal area may make it difficult to distinguish BD from CD. Focal colitis has been documented in colorectal biopsies obtained from patients with BD but without visual or clinical evidence of rectal involvement demonstrable by either endoscopic or radiologic examinations.

The abdominal complaints that lead to surgical intervention are abdominal pain (92%), an abdominal mass (21%), and/or melena (17%) [3]. Histopathologically, Behçet's ulcers contain nonspecific chronic inflammation, and the submucosal connective tissue appears disrupted. Granulomas are absent. The mucosa around the ulcers is usually normal in appearance [20]. The presence of focal ulcers, fistulae, and strictures and the ileocecal location may mimic CD. Since the histologic features are nonspecific, one must rely on the clinician to suggest the diagnosis. Just like the case reported by Jarrahnejjad et al., the patient can be misdiagnosed as having ulcerative colitis until pathological diagnosis of vasculitis is determined [20, 24]. However, Kim et al. suggest that inflammatory bowel diseases and Behçet's disease may be closely related and part of a spectrum of disease rather than distinct disease entities [25]. 

There may also be perienteric infiltration, mild lymphadenopathy, mesenteric vascular engorgement, mild splenomegaly, and minimal ascites. Complications of bowel perforation and peritonitis occur more commonly in patients with a thickened bowel wall and severe perienteric infiltration rather than those with polypoid lesions. Although many patients with BD have abdominal pain similar to appendicitis, the distinction can often be made with CT scanning. MRI also demonstrates bowel wall thickening and increased enhancement as well as extraluminal manifestations such as mesenteric infiltration around the involved bowel. Indium-111-labeled leukocytes can demonstrate localization in an area of inflammation [2]. 

### 2.1. Pancreas

A patient with BD can develop features consistent with either acute or chronic pancreatitis. Other etiologic factors for pancreatitis such as gallstone, alcohol, trauma, hyperlipidemia, and an infectious disease process should be ruled out before accepting the case as being related to BD. Biochemical evidence of pancreatitis can occur in the absence of either sonographic or computer tomographic findings and is a relatively common occurrence in cases of BD [2]. Acute pancreatitis may respond to corticosteroids.

### 2.2. Liver

The most common hepatic complication of BD is Budd-Chiari syndrome (BCS). BCS is an uncommon form of portal hypertension caused by obstruction of the hepatic venous outflow usually due to thrombi in the hepatic veins. The main clinical features are hepatomegaly, leg edema, ascites, and venous dilatation over the trunk. The radiologic and liver biopsy findings confirm the diagnosis and provide information for the selection of appropriate medical, interventional, or surgical therapies. Because vasculitis is a major pathologic component of BD, hepatic vein vasculitis and BCS occur often in cases of BD. However, with no histologic evidence of vasculitis, a case of bile duct inflammation resembling small duct primary sclerosing cholangitis has been also reported. 

The study of Bayraktar et al. is the largest series in the literature, consisting of 14 cases of BD-associated BCS. Based on this experience, it appears as if BCS is a relatively frequent complication of BD that accounts for 42.4% of the cases of BCS with recognizable underlying disease in Turkey. In most patients with BD and BCS, the IVC, as well as the hepatic veins, is involved. The clinical course of BCS in BD is poor [3]. The major determinant of survival is the extent of the vascular thrombosis in the IVC. BD may also contribute to development of cavernous transformation of the portal vein, portal vein thrombosis with splenomegaly, and superior vena cava thrombosis. 

The other hepatobiliary conditions reported to occur in cases of BD are hepatomegaly due to fatty liver or congestion, cirrhosis, acute hepatitis, chronic hepatitis, cholelithiasis, acute cholecystitis, toxic hepatitis, hepatic abscess, primary biliary cirrhosis, and hepatocellular carcinoma. Acute liver failure and rapid death were found in one-third of patients in one series. Münke et al., suggested an association between BD and chronic hepatitis C [26]. Subsequently, Oğuz et al. reported a 0.45% prevalence of anti-HCV antibodies in a series of 224 cases of BD in Turkey [27]. They stated that this finding is similar to the prevalence of anti-HCV that occurs in healthy subjects and is much lower than the prevalence in hemodialysis patients (51%) [3]. Rare cases include a hepatic artery aneurysm causing hemobilia as well as pylephlebitis and septic thrombophlebitis of the portal vein. Type AA amyloidosis complicates BD and presents most often as diarrhea and malabsorption. It mainly involves the GI tract and the kidneys with proteinuria that progresses to nephrosis and renal failure. It has a 50% mortality rate after an average duration of 3.4 years.

## 3. Differantial Diagnosis

Behçet's disease should be considered in the differential diagnosis in patients with recurrent orogenital ulcerations and enterocolitis with a nonspecific histological feature [28].

### 3.1. Crohn's Disease

The diagnosis of Behçet disease requires the presence of recurrent oral ulceration. Other helpful clinical features include ocular involvement, arthritis, erythema nodosum, and recurrent genital ulceration. Unfortunately, some of these may also be present in CD [20]. While granuloma formation is a pathologic hallmark of CD, it is not a characteristic lesion of BD just like cobblestoning and there is less inflammation surronding ulcer in BD compared to CD [12]. Longitudinal ulcers and granulomas tend to be uncommon in Behçet's colitis, Naganuma et al. reported intestinal Behçet's disease with longitudinal ulcers and noncaseating granuloma [29]. The most characteristic pathologic feature of BD is the finding of deep ulcers associated with vasculitis, usually a venulitis [12]. The intestinal wall is of normal thickness, unlike the rigid, narrowed segments seen in CD. In addition, fistula formation and intestinal perforation tend to occur early in BD as compared to CD where they occur in a later course of the disease. Free perforation is rare in cases of CD but can occur in BD. Korman et al. found that in CD, but not in BD, enteroclysis, a radiologic examination of choice in the detection of pathologic changes in small bowel that shows the site and extension of the involvement, mucosal abnormalities, complications, and recurrences, findings were more severe, and cases generally were in the advanced stage when compared with the duration of both diseases [9]. Scalloping, ulceronodular patterns, and complications such as abscess formation are not observed in intestinal BD, therefore certain EC findings are helpful in differentiating BD from CD.

### 3.2. Steroid and Nonsteroid Anti-Inflammatory Drug-Induced Damage

Steroid and nonsteroid anti-inflammatory drug-(NSAID-) induced damage involves primarily proximal large intestine and occurs in various forms including colitis, colonic ulcers, pseudomembranous colitis, collagenous colitis, bleeding, and perforation, while aphthous ulceration is very rare. NSAIDs may occasionally cause small intestinal perforation, ulcers, and strictures that require surgery. The patient's clinical history is important since many patients with BD use steroids to control their disease. The ulcers that occur in cases of BD differ from steroid-induced ulcers both in size and appearance. Steroid-induced ulcer is usually single while the ulcers of BD seen in long-term steroid treatment are characterized by multiple perforations. In one study, the frequency of intestinal perforation in patients with BD on steroids was 41%. Also in cases of BD without a history of steroid use, intestinal perforation can occur in up to 33% of the patients [12].

### 3.3. Ulcerative Colitis

The ileocecal region is the most frequently involved location in colonic BD. However, in ulcerative colitis the disease usually starts in the rectum and moves to the right colon, and also the ulcers are deeper in BD than ulcerative colitis [12]. Furthermore, HLA-B51 has never been reported in association with ulcerative colitis. Rather significant association with B52 and DR2 with UC has been reported [30].

### 3.4. Amebiasis

Differantion of amebiasis and BD is important in the Middle East where both were frequently occur. Endoscopically, the disease is diffuse in both conditions, and the mucosa is hyperemic and is characterized by deep ulcers. But the presence of an amoeba in a fresh stool specimen is the best way to differentiate the two conditions [12]. 

## 4. Prognosis and Treatment

Prognosis of BD runs a chronic, unpredictable course with exacerbations and remissions which decrease in frequency and severity over time. Death is mainly due to major vessel disease and neurological involvement. The prognosis is poor for young males. Treatment of BD is usually palliative and symptomatic. The preferred treatments are combined drug therapy with any or all of the following: steroids, NSAIDs, immunosuppressives, and cytotoxic agents [20]. Complete remission is achieved in 38% of patients with intestinal BD after 8 weeks of medical treatment. Surgery is the other modality of treatment. Recurrence occurs in 49% of patients in 5 years, especially those with intestinal perforation or fistula formation. Recurrent lesions ocur at or near the anastomoses in 1% of patients. Of those who undergo surgery, 75% recur within 2 years and are associated with a higher rate of complications such as ocular and ileal lesions than the nonsurgical group. The incidence of postoperative recurrence is less in those with normal intraoperative endoscopy than those with lesions seen. High rate of recurrent disease is observed especially in patients of Western or Chinese origin [15]. The histologic features of recurrent disease resemble the primary disease.
